# Prevalence and Factors Associated with Thinness in Rural Polish Children

**DOI:** 10.3390/ijerph17072368

**Published:** 2020-03-31

**Authors:** Agnieszka Suder, Paweł Jagielski, Beata Piórecka, Małgorzata Płonka, Karol Makiel, Matylda Siwek, Iwona Wronka, Mariusz Janusz

**Affiliations:** 1 Department of Anatomy, University of Physical Education, 31-571 Krakow, Poland; 2 Department of Nutrition and Drug Research, Jagiellonian University Medical College, 31-531 Krakow, Poland; 3 Department of Tourism and Regional Studies, Pedagogical University of Krakow, 30-084 Krakow, Poland; 4 Department of Anthropology, Institute of Zoology and Biomedical Research, Jagiellonian University, 30-387 Krakow, Poland; 5 Independent Researcher, 32-445 Krzyszkowice, Poland

**Keywords:** risk factors, BMI, physical activity

## Abstract

A lot of attention has been focused on obesity, however, the other extreme—thinness—may lead to inhibition of physical and intellectual development. The aim was to assess the prevalence of thinness and determine the associated factors in children from rural populations. We used data from the cross-sectional sample of 3048 children, examined in schools from a district in southern Poland. The sample included 89% of the district departments, and included a proportion of rural and small town populations—a representative one for the region. Thinness was determined based on the criteria proposed by Cole and Lobstein. Biological, sociodemographic and lifestyle factors were analysed. The odds ratio (OR) and 95% confidence interval (CI) were calculated using logistic regression analysis. The prevalence of thinness was 11.5% in boys and 13.5% in girls. In the younger group, it was similar in boys and girls at 11.8%; whereas in the older group, it was 11.1% and 14.5%, respectively. The prevalence of thinness Grade 3 in girls was two times higher than in boys. The increased index of leisure time physical activity was connected with thinness in prepubertal boys. The mechanism determining the development of thinness is very complex and further exploration of this trend is recommended.

## 1. Introduction

In developed countries, there is abundant information on the epidemic of childhood obesity, but there are fewer studies devoted to the other extreme—thinness. Despite the rise in mean Body mass index (BMI), more children and adolescents worldwide are moderately or severely underweight than obese [1]. According to WHO, both too low and too high a body weight are on the list of ten factors that are the most threatening for human health. It is estimated that currently there are more than 1.5 billion people with excessive body mass worldwide, 522 million of whom are obese; whereas about 15% of society are affected by body mass deficiency [2]. Societies have been experiencing only one of these health problems for many years. Developing countries were dominated by an underweight population, while the developed ones had problems with an overweight population. At present, both conditions coexist, and both types of countries are facing new problems which are very different to the former ones [2]. On the basis of pooled global data for children and adolescents aged 5–19 years, in 2016, 192 million children worldwide were moderately and severely thin, while, in the same year, 124 million children were obese [3]. The problem of thinness concerns countries with different levels of socioeconomic status and is attributed to medical, social and economic issues [2,3]. A poor diet can be a result of several factors such as inadequate food and beverage intake, lack of available food and drink, chronic food shortage, chronic hunger, starvation and food insecurity [4]. Underweight and weight loss can be caused by parasites (insects, fungi, viruses and helminths or parasitic worms) that attack an individual’s body. Thinness in children can also result from wrong eating habits, such as early feeding of solid foods while withholding breast milk and formula, lack of essential nutrients (essential amino acids, essential fatty acids, critical vitamins and minerals) in food given to a child, insufficient and irregular feeding of an infant, nonexposure of the child to adequate sunlight to make vitamin D necessary for proper bone development, and the early introduction of a child to weaning and complementary foods that consist of adult foods (such as tea, high fibre foods) containing antinutrient (phytates, tannins) that decrease the absorption of vitamins and minerals. Some children can also be underweight because of fatigue and emotional issues such as stress and anxiety [4]. In European countries, thinness is more prevalent among adolescent girls and young females, potentially because of their desire to attain an image of beauty represented by thinness as advertised by the fashion industry [4,5]. 

The relatively rapid transition from underweight to overweight and obesity in low- and middle-income countries and the dual burden of body weight are of public health importance because policies targeted at controlling obesity may have undesirable effects on the thinnest individuals [6,7,8]. Improper macronutrient and micronutrient intake leads to increased infections, cardiac and renal dysfunction, anaemia, osteoporosis, delaying the process of growing up and developing, and increased mortality [9]. Too low a body weight results in malfunctioning of the reproductive system, menarche delays or disappearance of existing menarche [10]. During reproductive maturity, women’s body weight deficiencies may cause an improper course of pregnancy, inhibition of intrauterine growth of the foetus, complications and premature delivery [10,11]. Another significant result of body mass deficiencies is insufficient amounts of vitamin D, which may lead to a lowered bones density. This problem most frequently affects young girls [4]. In children, thinness could be a sign of malnutrition attributed to unhealthy eating behaviours [4]. The main negative consequences of undernourishment in children comprise inhibited growth and development in the form of stunting and wasting in children, low lean body mass or low muscle mass, failure to thrive, developmental delays in children, a weakened or compromised immune system that makes a person prone to infections, fragile bones, poor self-control, poor sleep, a relative energy deficiency in sports [4].

In Poland, which at the turn of the 20th and 21st centuries experienced deep economic, social and political transition causing important changes in lifestyle, a relatively high prevalence of thinness is still present [12,13,14]. In 2005, body mass deficiency among teenagers aged 13–15 years in Poland was observed in 4.2% of secondary school students (4.6% in boys, 3.8% in girls), being slightly more frequent in rural students (4.4%) than in city dwellers (4%) [15]. Currently, in Poland, the prevalence of thinness in girls as a nationally representative sample of children is close to the prevalence of overweight and obesity amounting to 13.6% [16]. The period of the 1980s was the time of economic collapse, goods rationing and difficulties in food supply. The lifestyle in the 1990s was improved by Polish accession to the European Union in 2004, and despite being more intensified in cities, these changes also affected rural Poland. The rural environment generally features lower levels of education of the inhabitants, greater poverty, lower health consciousness, and it may feature complex problems regarding access to health care [17]. However, the biological effects of political transformation and living standards can vary and depend on the population being examined [18].

The aim of the work was to estimate the prevalence of thinness in boys and girls in a rural environment and to determine factors which significantly increase the probability of the occurrence of thinness in rural children from a selected administrative district located in southern Poland.

## 2. Materials and Methods 

The study material includes a cross-sectional sample of 3048 children (1492 boys and 1556 girls) aged 7–12 years; they were primary schools students examined within the years 2009–2011 in schools of the Myślenice district located in the southern part of Poland, near the city of Krakow. A detailed description of the children according to the average level of somatic features and the prevalence of abdominal obesity has been presented elsewhere [19]. The sample included 89% of the district’s smaller administrative departments, and it also included a proportion of the rural population and the population from small towns [20], therefore it can be treated as representative for the region. The tests were performed after obtaining written agreement from school headmasters and the children’s legal guardians. The protocol was conducted according to the ethical principles stated in the Helsinki Declaration (1975).

For the purpose of this study, body height (Ht) and body weight (Wt) were used. Height was measured without shoes, in the standing position to the nearest 1 mm, with the head in the Frankfurt plane, using an anthropometer. Weight was obtained in the standing position with a standardized medical scale with an accuracy of 100 g. Anthropometric examinations were performed by anthropologists. Measurements were taken before noon in separate rooms. Body mass index (BMI; kg/m^2^) was calculated as weight (kg) divided by squared height (meter). International standards elaborated by Cole and Lobstein [21] were used for the assessment of the prevalence of thinness. Thinness Grade 1, 2, and 3 correspond to the BMI 17 to <18.5, 16 to <17 and <16 kg/m2 at the age of 18 years, respectively.

The children’s lifestyles and their families’ socioeconomic status were investigated through survey questionnaires [22,23]. The questionnaires filled in by children and their parents were returned by 71.6% of the participants and further analyses included diminished numerical strength. 

The questionnaires were used to assess place of residence, parents’ educational level, frequency of consuming selected food products by children, regularity of meals, and also the number of hours of physical activity during the children’s leisure time and children’s participation in selected forms of physical activity. The place of residence, including small towns and rural departments, were determined according to the Central Statistical Office [24] in accordance with the December 15, 1998 Council of Ministers Directive establishing the official national register of the territorial division of Poland. Parents’ educational level was defined as low (elementary or basic vocational, secondary comprehensive or technical and high (incomplete university or university). Frequency of consuming selected food products was defined based on the declared number of days in a week: vegetables ≤ 4, fruit ≤ 4, sweets > 4. Regularity of breakfasts (at least a little meal, e.g., a sandwich or cereal with milk consumed before going to school and was assessed (yes/no)). 

Data regarding leisure time physical activity (such as cycling, roller skating, football, basketball, volleyball and others) with a determined number of days per week and time periods per day were used to assess the leisure time physical activity index in the participating children. According to the Compendium of Physical Activities [25], each form of activity received a certain number of metabolic equivalents (METs) and the measure of physical activity was the sum of METs obtained from particular physical activities. The metabolic equivalent of a task (MET) is an objective measure of the ratio of the rate at which a person expends energy, relative to the mass of that person, while performing some specific physical activity compared to a reference, set by the convention at 3.5 mL of oxygen per kilogram per minute, which is roughly equivalent to the energy expended when sitting quietly. The total assessment of an activity was calculated based of the MET formula expressed as MET h/week as follows: value of 3 METs (intensity of slow cycling) x 0.5 h (duration per day) x 2 times per week (frequency) = 3 MET × h−1 × week−1. Two groups of leisure time physical activity index based on the characteristics of the METs values distribution were distinguished: low and high.

## 3. Results

The anthropometric description of the study sample is presented in Table 1. Statistically significant differences between sexes were found in relation to body height in eight- and nine-year-olds, and weight and BMI in 12-year-olds.

The prevalence of thinness in rural boys and girls in the whole examined sample was 11.5% and 13.3% (Table 2), respectively; however, statistically significant sex differences refer to the older age group (10–12 years) where the prevalence of thinness was significantly higher in girls (14.5%) when compared to boys (11.1%) (Table 3). 

A more thorough analysis of the prevalence of thinness shows its rising tendency with the girls’ age, from 9.8% for the youngest girls to 16.1% for 12-year-old girls; whereas in the male group, the tendency was opposite. The prevalence of thinness Grade 3 in girls was two times higher than in boys (Table 2).

Table 4 presents a statistical analysis of the biological, sociodemographic and lifestyle risk factors associated with thinness in rural girls and boys. Sociodemographic variables such as the place of residence and parents’ education were not statistically significant risk factors, nevertheless, living in a rural environment indicated higher odds ratio values. From among the lifestyle factors connected with eating habits, no significant correlations were found. Taking into consideration the lifestyle factors connected with physical activity, a high index of leisure time physical activity in boys turned out to be a significant factor of thinness, doubling the risk in boys in prepubertal age. 

Deeper analyses of the frequency of physical activity per week showed that boys with thinness undertook weekly physical activity more rarely, however, the activity lasted longer compared to the other boys (Table 5). Diversity in undertaking weekly physical activity or daily hours of physical activity were not confirmed among the girls with thinness in comparison to the remaining participants (Table 5).

## 4. Discussion

Thinness was found in about 12.4% of rural Polish children and adolescents, and in this study, the prevalence of thinness was slightly higher in girls in comparison to boys. Furthermore, the dependencies are similar to those of the population thus making this a nationally representative sample of Polish children [16]. This result is also supported by international research [26,27,28]. Age differences mainly refer to the older group where the prevalence of thinness was significantly higher in girls (14.5%) than in boys (11.1%), also confirming the tendency of girls’ thinness to increase with age [29,30]. Generally, the prevalence of thinness in Polish children is higher than in other European countries or Australia and USA, where thinness among children and adolescents is about 4%–9% [26,28,31,32]. The increasing percentage of children, especially older girls, with body weight deficiencies observed between 1977 and 2004 [8] in the Polish population is also alarming. A similar tendency in adolescence was also found in the Czech Republic [33], Sweden and Greece [26]. In France in 2009–2013, the frequency of thinness increased from 12% to 16.7% among girls, while in boys, the growth was insignificant (from 7.1% to 7.3%) [34]. The causes of such a tendency may lie in sociocultural determinants, the influence of the media in making the slim silhouette fashionable and more active weight-control behaviour in pubertal girls independently of socioeconomic status [5,13,35,36]. Psychological factors, such as self-esteem and sense of purpose, body image and emotional status may also play a role, with poor psychological health being associated with thinness among girls in the industrialised world [37,38]. The changes may also result from trends in morphological changes during puberty and still present a positive secular trend associated with body height [14].

In a representative sample of Polish children [16], it was demonstrated that the prevalence of thinness was highest among boys and girls of mothers whose educational level was low, and in families which declared low income per capita. The result of this study does not confirm a significant relation between parents’ educational level and thinness, nevertheless, a lower educational level of the father was connected with occurrence of thinness both in sons and daughters. Generally, higher parental education has been shown to work as a protective shield for thinness, as higher socioeconomic status and higher parental income decreased the risk of thinness in children [39]. 

The remaining sociodemographic variables, such as place of residence, did not confirm that the rural environment was a more significant risk factor of body mass deficiency occurrence in children in comparison to the environment of small towns in southern Poland. This may be linked to the fact that the administrative units of the Myślenice district are relatively economically well developed, for example, tourist attractions (the Raba river valley area surrounded by the Beskid mountains) improve the situation of people in the region offering possibilities to earn more money. The region hosts highly specialised companies in the field of trade, production, building, transport and one fourth of the population are farmers [20]. The test results on children and the youth development from rural/urban areas, despite sometimes remarkable inequalities in education, professional qualifications or earnings, show that the differences in biological features may disappear [40]. In addition, the tests that have been recently conducted in Poland confirm that the rural–urban distances in biological development have significantly decreased [41].

The results of worldwide research confirm that regular family meals prevent eating disorders [42]. Adolescents who eat breakfast regularly have a normal BMI, which can vary for those who eat breakfast intermittently [43]. Adolescent girls have pressure to be thin and may try to achieve this by skipping meals. Most of the surveyed girls declared eating regular breakfasts but the variable was not significant. The influence of the other variables which described the frequency of consuming vegetables, fruit and sweets, determined based on declared numbers of days per week turned out to be insignificant.

This study found boys active in leisure time (more than 2 h) to be more likely to be thin in comparison to their physically inactive peers. The examined group of boys (aged 7–12 years) was in the early stage of ontogenesis in relation to maturation and body structure formation. Muscle mass that affects the BMI values is not differentiated in boys at this age. Its general differentiation starts after the age of 12 when fibres intensively grow and get thick. This connection with physical activity during leisure time may also result, in the case of rural children, from additional energy expenditure during household/farm work. The opposite results were found in the study of Mason et al. [27] which showed inactive boys and girls to be more likely to be thin. Furthermore, in a Swedish study, low physical activity during leisure time was associated with thinness in both genders [30]. Studies regarding the associations between thinness and physical activity levels are sparse, but the authors observed that thinness coexists with lower levels of physical activity in boys and lower interest in this form of activity in thin children. The low muscle mass markedly affects the ability to perform physical exercise, and from the tests of Artero et al. [29] and Castro-Pinero et al. [44], it was shown that body weight deficiencies also determine the level of health-related fitness. The study of Malina and Katzmarzyk [45] demonstrates that school-aged children of marginal to poor nutritional status have a decreased total daily energy expenditure and energy expended in physical activity, which is partially connected with the smaller body size. The reduced muscle mass associated with chronic and mild-to-moderate undernutrition influences the strength, performance, PWC_170_ (physical working capacity at a heart rate of 170 beats per minute) and VO_2max_ (oxygen uptake) in samples of school-aged children. The extreme lower levels of fitness are generally proportional to the reduced stature and body mass of children and adolescents living under conditions of chronically marginal nutritional circumstances [46]. 

The obtained results, which indicate a high percentage of children with body weight deficiencies, are relevant when connected to the theory that undernutrition in early life might promote obesity in adulthood. This is reflected by the studies by Barker [47] that show that thin born adults developed abdominal obesity and other features strongly related to a metabolic syndrome. In addition, one of the postponed effects of privation that affects pregnant women is a permanent change in foetus gene expression, which is responsible for the development of tissue resistance against insulin [48].

A recent paper documents that worldwide, the prevalence of thinness remains high, even though the prevalence of obesity is increasing in many regions of the world. The worldwide rate of increase in the prevalence of obesity in children and adolescents is greater than the rate of decline in undernutrition [2]. Most health-related programs in developed countries are oriented towards obesity prevention while the problem of thinness is usually not addressed. The mechanism determining the development of thinness is very complex and further exploration of this trend is recommended. 

## 5. Conclusions

In conclusion, thinness was found in about 12.4% of rural Polish children and adolescents, and age differences mainly refer to the older group where thinness prevalence was significantly higher in girls (14.5%) than in boys (11.1%). The rural environment was no more a significant risk factor of thinness in children in comparison to the environment of small towns, which confirms the general trend of decreasing rural–urban distances in biological development in Poland. This study found boys active in leisure time to be more likely to be thin in comparison to their physically inactive peers, which is connected with the their early stage of ontogenesis (7–12 years) in relation to maturation and body structure formation. The mechanism determining the development of thinness is very complex and further exploration of this trend is recommended. 

## Figures and Tables

**Table 1:** Statistical characteristics of height (Ht), weight (Wt) and body mass index (BMI) in rural boys and girls.

Age	Ht (mm)						Wt (kg)					BMI				
	n	$\bar{x}$	SD	Me	Min	Max	$\bar{x}$	SD	Me	Min	Max	$\bar{x}$	SD	Me	Min	Max
Boys																
7.0	110	1256.9	54.3	1256.5	1119.0	1378.0	25.8	5.3	24.6	18.0	42.0	16.1	2.5	15.6	11.3	24.2
8.0	302	1297.2 *	58.8	1294.0	1131.0	1503.0	27.8	5.9	26.5	11.7	57.7	16.4	2.3	15.9	12.4	27.6
9.0	299	1357.7 *	60.5	1356.0	1210.0	1505.0	32.1	6.5	31.0	20.0	55.0	17.4	2.8	16.7	12.8	26.4
10.0	306	1405.7	63.8	1399.0	1199.0	1611.0	35.4	8.6	33.3	21.9	70.3	17.6	3.2	16.7	11.9	29.1
11.0	257	1457.4	64.4	1455.0	1299.0	1698.0	38.4	8.3	36.7	22.1	71.3	18.0	2.9	17.1	12.9	27.4
12.0	227	1515.2	69.9	1510.0	1308.0	1693.0	43.9 *	11.4	41.2	25.9	88.0	18.9 **	3.9	17.9	13.0	36.8
Girls																
7.0	121	1239.7	59.3	12420	1095.0	1395.0	25.0	5.8	24.0	14.8	54.5	16.2	2.6	15.9	12.1	28.0
8.0	306	1285.7	58.2	1285.0	1116.0	1425.0	27.3	5.2	26.4	16.0	44.9	16.4	2.4	15.9	8.0	23.7
9.0	274	1338.8	58.3	1340.0	1200.0	1521.0	31.5	7.7	29.6	18.1	70.6	17.3	3.2	16.6	11.5	30.5
10.0	303	1404.0	65.0	1406.0	1216.0	1620.0	34.7	7.7	33.4	21.3	64.6	17.6	2.9	17.0	11.2	28.3
11.0	308	1460.0	69.5	1458.0	1256.0	1666.0	38.2	8.7	36.6	23.4	76.8	17.7	3.1	17.0	9.3	31.2
12.0	254	1515.5	62.3	15220	1308.0	1673.0	42.6	9.3	41.7	25.7	80.7	18.4	3.1	17.8	12.9	30.1

n, sample size; $\bar{x}$, mean; SD, standard deviation; Me, median; Min, minimum; Max, maximum; The boys’ mean measurement significantly different from girls’ at *p* < 0.05 * test t; ** Mann–Whitney test.

**Table 2:** The category of thinness in rural boys and girls.

	Boys		Girls		Boys and Girls	
BMI category	n	%	n	%	n	%
Thinness Grade 1	141	9.4	160	10.2	301	9.8
Thinness Grade 2	25	1.7	35	2.3	60	2.0
Thinness Grade 3	6	0.4	13	0.8	19	0.6
Thinness all grades	172	11.5	208	13.3	380	12.4

Thinness Grade 1, 2 and 3 correspond to the BMI 17 to <18.5, 16 to <17 and <16 kg/m^2^ at the age of 18 years, respectively, (Cole and Lobstein [21]).

**Table 3:** The prevalence of thinness in rural boys and girls according to age category.

	Boys		Girls		Chi-Square Test
Age	n	%	n	%	*p*
7.0	21	19.1	16	13.2	0.2245
8.0	37	12.3	30	9.8	0.3351
9.0	26	8.7	37	13.5	0.0660
10.0	37	12.1	32	10.6	0.5513
11.0	23	8.9	52	16.9	0.0056 *
12.0	28	12.3	41	16.1	0.2344
7–9	84	11.8	83	11.8	0.9879
10–12	88	11.1	125	14.5	0.0444 *
Total	172	11.5	208	13.3	0.1255

* Frequencies of boys significantly different from the frequencies of girls at *p* < 0.05 (chi-square test).

**Table 4:** Factors associated with thinness (BMI) in rural boys and girls.

Variables	Category	%		BMI			
				Boys		Girls	
		Boys	Girls	OR	95% CI	OR	95% CI
Child’s age	7–9	46.4	44.7	1.00	0.69–1.32	1.00	0.88–1.61
	10–12	53.6	55.3	0.95		1.19	
Place of residence	small town	28.8	26.3	1.00	0.81–1.75	1.00	0.66–1.32
	rural department	71.2	73.7	1.19		0.94	
Father’s education	low	75.7	74.3	1.00	0.59–1.45	1.00	0.64–1.48
	high	24.3	25.7	0.93		0.97	
Mother’s education	low	58.4	59.6	1.00	0.81–1.94	1.00	0.68–1.49
	high	41.6	41.3	1.25		1.07	
Vegetables consumption (≤4 days a week)	no	26.6	28.9	1.00	0.59–1.88	1.00	0.76–1.96
	yes	73.4	71.1	1.05		1.22	
Fruit consumption (≤4 days a week)	no	46.7	47.1	1.00	0.50–1.40	1.00	0.52–1.29
	yes	53.3	52.9	0.83		0.82	
Sweet food consumption (>4 days a week)	no	66.5	66.9	1.00	0.72–2.03	1.09	0.70–1.80
	yes	33.5	33.1	1.21		1.12	
Regularity of breakfasts	no	5.4	5.7	1.00	0.27–3.31	1.00	0.64–11.7
	yes	94.6	94.3	0.94		2.75	
Leisure time physical activity index	low	65.5	69.8	1.00	1.21–7.16 *	1.00	0.57–5.68
	high	34.5	30.2	2.95		1.80	

OR, odds ratio; CI, confidence interval; * significance at *p* < 0.05.

**Table 5:** The prevalence of frequency and the duration of physical activity in rural boys and girls with thinness (BMI < 18.5) and without (BMI ≥ 18.5).

	BMI < 18.5	BMI ≥ 18.5	Chi-Square Test
Boys	%	%	*p*
Frequency of physical activity			
Up to 3 times per week	77.3	64.8	0.0423 *
More than 3 times per week	22.7	35.2	
Duration of physical activity per day			
Up to 2 h	88.4	95.1	0.0248 *
More than 2 h	11.6	4.9	
**Girls**	**%**	**%**	***p***
Frequency of physical activity per week			
Up to 3 times per week	77.6	71.9	0.8961
More than 3 times per week	27.4	28.1	
Duration of physical activity per day			
Up to 2 h	95.2	97.2	0.3267
More than 2 h	4.8	2.8	

* significance at *p < 0.05* (chi-square test).
